# Stay-At-Home Orders Are Associated With Emergence of Novel SARS-CoV-2 Variants

**DOI:** 10.7759/cureus.13819

**Published:** 2021-03-11

**Authors:** Ricardo A Zimerman, Flavio A Cadegiani, Rute Alves Pereira e Costa, Andy Goren, Bruno Campello de Souza

**Affiliations:** 1 Infectious Diseases, Hospital da Brigada Militar, Porto Alegre, BRA; 2 Clinical Director, Applied Biology Inc, Irvine, USA; 3 Clinical Endocrinology, Federal University of São Paulo, São Paulo, BRA; 4 Endocrinology, Corpometria Institute, Brasilia, BRA; 5 Dermatology, Applied Biology Inc, Irvine, USA; 6 Management Sciences, Federal University of Pernambuco, Recife, BRA

**Keywords:** covid-19, sars-cov-2, e484k mutation, sars-cov-2 variants, pandemic

## Abstract

Background

While public health strategies to contain the current coronavirus disease 2019 (COVID-19) pandemic are primarily focused on social distancing and isolation, emerging evidence suggest that in some regions social isolation failed to lead to further decrease in the number of COVID-19 deaths in the long run. This apparent paradox was particularly observed in the northern region of Brazil, in the state of Amazonas. We hypothesized that the emergence of new severe acute respiratory syndrome coronavirus 2 (SARS-CoV-2) mutations, leading to more transmissible and pathogenic variants, could explain the lack of further reductions in COVID-19 new cases and related deaths in some regions. Our objective is to determine if social isolation is associated with the emergence of new SARS-CoV-2 variants, particularly the P.1 lineage and E484K mutants, in Brazil and in the state of Amazonas.

Materials and methods

We assessed the prevailing SARS-CoV-2 genomes present in Brazil available on the GISAID (Global Initiative on Sharing All Influenza Data) database collected between June 1, 2020, and January 31, 2021. Data regarding demographics, lineage, and prevalence of P.1 lineage and E484K mutations were obtained. Social isolation was measured using the Social Isolation Index (SII), which quantifies the percentage of individuals that stayed within a distance of 450 meters from their homes on a given day, between February 1, 2020, and January 24, 2021. The number of daily COVID-19 deaths was obtained from the Brazilian Ministry of Health (OpenDataSUS, 2021) between March 12, 2020, and January 10, 2021. SII was correlated with the prevalence P.1 lineage and E484K mutations in the eight following weeks. All univariate associations were estimated using the Spearman Correlation Index. 3D surfaces were employed to reflect the relationship between time, social isolation, and prevalence of genomic variants simultaneously.

Results

A total of 773 and 77 samples were obtained in Brazil and in the Amazonas state, respectively. In the state of Amazonas, SII on a given week was positively, significantly, and moderately or strongly (r > 0.6) correlated with the prevalence of both P.1 lineage and other E484K variants in the six following weeks after the SII on a given week. Conversely, in overall Brazil, correlations between SII and P.1 lineage and E484K variants were weaker and shorter, or negative, respectively. When SII was below 40%, P.1 lineage or E484K variants were not detected in the following weeks. When SII was above 40%, apparently exponential positive correlations between SII and prevalence of both P.1 lineage and E484K variants were observed.

Conclusion

The results of this study indicate that SII above 40% is associated with the emergence of SARS-CoV-2 E484K variants and P.1 lineage in the state of Amazonas, which was not observed in overall Brazil.

## Introduction

Public health strategies to contain the current coronavirus disease 2019 (COVID-19) pandemic are primarily focused on social distancing and social isolation. However, emerging evidence suggests that social isolation might be positively associated with subsequent increases in the number of COVID-19 deaths in particular regions [1,2], possibly due to a combination of factors including viral transmission patterns and lack of access to medical care.

There is preliminary evidence that in the city of Manaus, the capital of the state of Amazonas, Brazil, a peculiar association was identified: the implementation of lockdown orders in two different periods were both followed by sharp rises in the number of COVID-19 hospitalizations and COVID-19 related deaths [3].

We hypothesized that the lack of further reductions in COVID-19 new cases and related deaths after social distance in particular circumstances and regions could be partially justified by the emergence of new, possibly more transmissible and pathogenic viral mutations.

In this communication, we attempt to assess if social isolation into small family or groups is associated with the emergence of new severe acute respiratory syndrome Coronavirus 2 (SARS-CoV-2) variants, particularly the P.1 lineage and E484K mutants, in Brazil and in the state of Amazonas. Increased rate of viral mutations would be of major concern, as those viruses have demonstrated ability of both immune evasion and stronger infectiousness and infectivity.

This article was previously posted to the ResearchGate preprint server in February 2021.

## Materials and methods

Population analyzed

For the present analysis, we considered the population of Brazil infected by the SARS-CoV-2 virus between June 1, 2020, and January 31, 2021, that underwent genetic sequencing of the virus.

Retrieval of the SARS-CoV-2 genome data

For the evaluation of the prevailing SARS-CoV-2 genomes present in Brazil and in the state of Amazonas, we selected all human related sequences available on the GISAID (Global Initiative on Sharing All Influenza Data) database [4] collected between June 1, 2020, and January 31, 2021. Data regarding demographics, lineage, and presence of the E484K mutation were obtained by specification in the search fields. For monthly counts, we considered the genomes with a collection date between the first and the last day of each month. For weekly counts, we considered seven-day periods starting from June 1, 2020.

Social Isolation Index

Social isolation was measured by the daily values of the Social Isolation Index (SII), which shows the percentage of individuals who stayed within a distance of 450 meters from their homes on a given day. Daily SII was collected from the In Loco© website [5] between February 1, 2020, and January 24, 2021, for Brazil and the state of Amazonas. The SII was based on the average of a 14-day period due to differences in isolation rates between weekdays and weekends.

Daily number of COVID-19 deaths

Number of daily COVID-19 deaths was noted between March 12, 2020, and January 24, 2021, through the official database of the Brazilian Ministry of Health [6]. We considered the actual, not the reported, date of death. The last 14 days of the series of data were removed from the analysis to avoid underestimation of the number of deaths due to the delay in the reports, i.e., we considered the daily number of COVID-19 deaths until January 10, 2021. The time series for the number of daily COVID-19 deaths were determined through 14-day based averages in order to avoid interference caused by differences in the quality of reports between weekdays and weekends.

Statistical analysis

We performed univariate associations between SII, viral mutations in the P.1 lineage and E484K variants, and number of COVID-19 deaths, which were estimated using the Spearman Correlation Index. We employed 3D surfaces to reflect the estimates for the relationship among time, SII, and prevalence of genomic SARS-CoV-2 variants in a concurrent manner based on the least squares with negative exponential smoothing. All calculations were performed using Microsoft Excel© 2019 (Microsoft, Redmond, WA, USA) and Tibco Statistica® 13.5 package (TIBCO Software Inc., Palo Alto, CA, USA).

## Results

Samples 

Table 1 shows the absolute number of samples obtained through the GISAID database with collection dates between June 1, 2020, and January 31, 2021. A total of 773 samples were obtained throughout the period encompassed by the present analysis in Brazil. Among them, 77 were from the Amazonas state.

**Table 1:** Number of genomic samples obtained in the state of Amazonas and in whole Brazil from June 2020 through January 2021.

	June	July	August	September	October	November	December	January
State of Amazonas	1	0	2	5	4	5	52	8
Brazil	124	156	48	32	78	202	96	37

Social isolation over time for the state of Amazonas and Brazil

**Figure 1:**
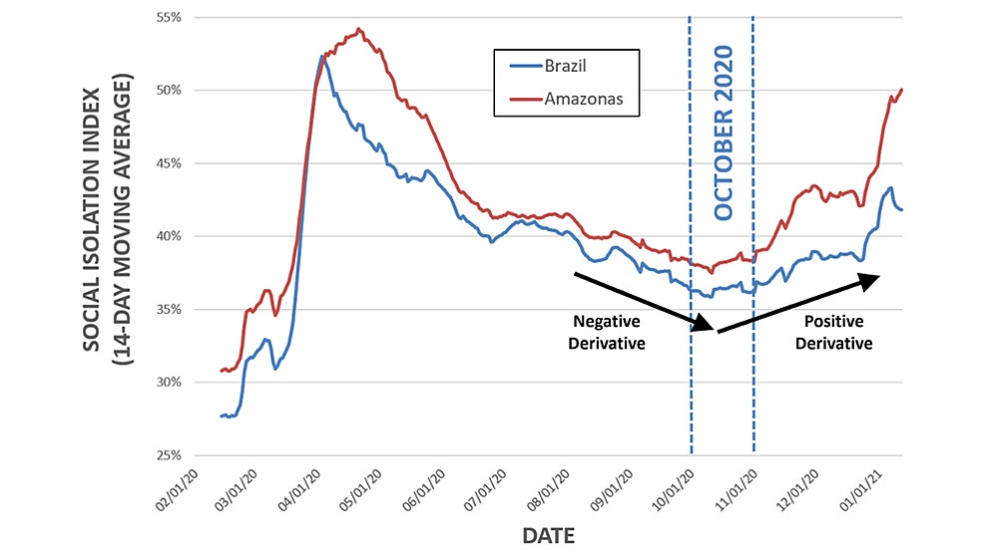
Social Isolation Index in the state of Amazonas and in Brazil.

Figure 1 shows the behavior of the SII in both the state of Amazonas and for the entire country of Brazil from February 2020 through January 2021. Both the State of Amazonas and Brazil experienced a fast growth in the SII after the Brazilian government announced social restrictive measures in March 2020, with a peak between April and May 2020, followed by a slow yet progressive reduction in SII until October, 2020. SII of the Amazonas state was higher than in Brazil throughout the period encompassed by the present analysis between February 1, 2020, and January 24, 2021.

COVID-19 deaths over time for Amazonas and Brazil

**Figure 2:**
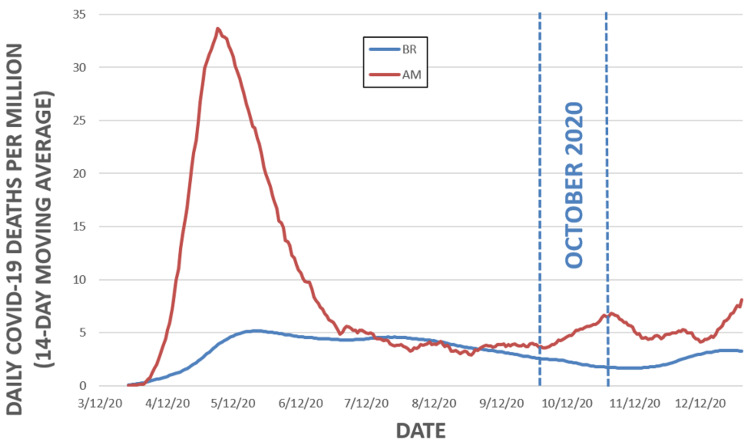
Daily number of deaths per million inhabitants in the state of Amazonas and in Brazil.

Figure 2 shows the progression of the number of daily deaths per million inhabitants both in the state of Amazonas and in Brazil. The relative number of daily COVID-19 deaths in the state of Amazonas was higher than in Brazil between April 2020 and July 2020 and between October 2020 and January 2021. The accumulated number of COVID-19 deaths per million inhabitants was 2,834.8 in the state of Amazonas, 2.95 times higher than in overall Brazil.

The prevalence of P.1 and E484K SARS-CoV-2 in the state of Amazonas and Brazil

**Figure 3:**
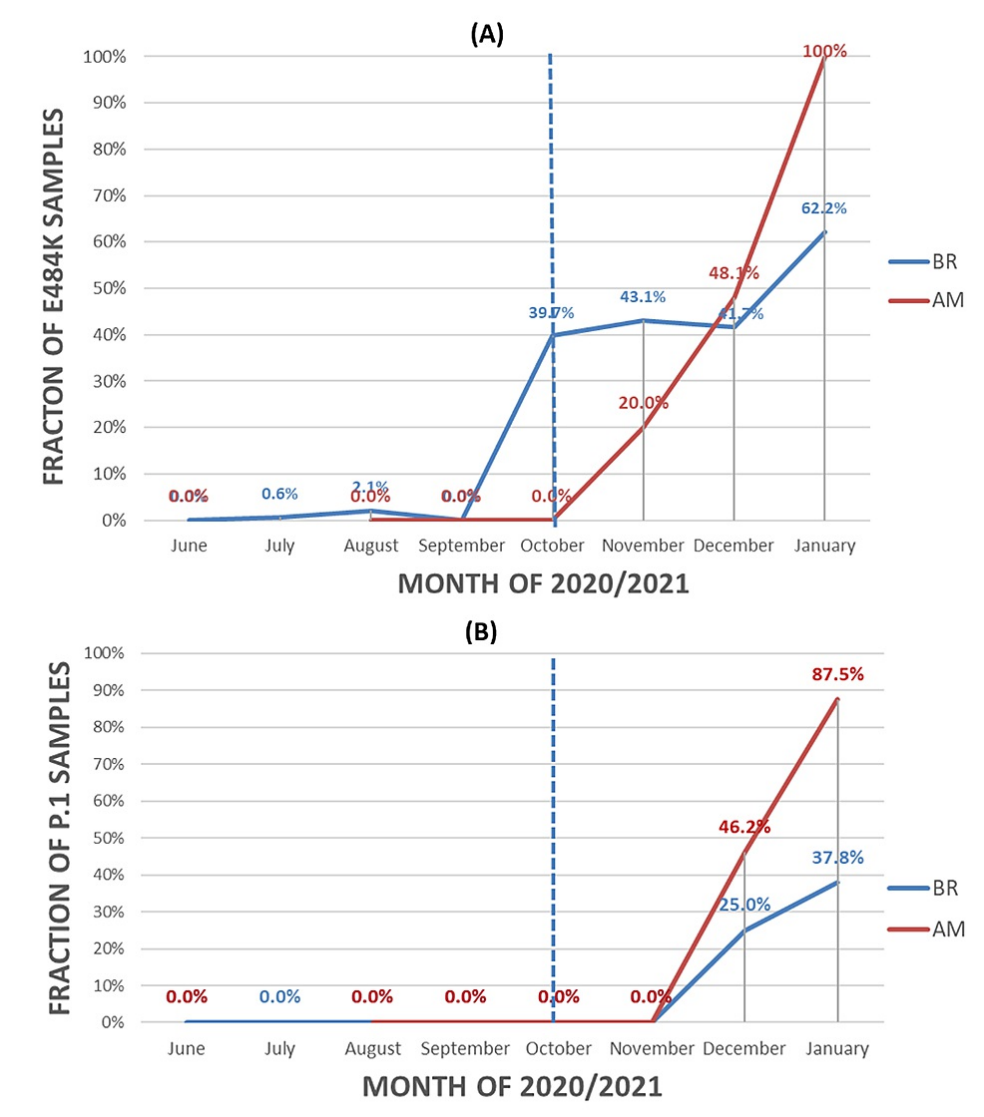
The percentage of all E484K variants (A) and P.1 lineage (B) in the state of Amazonas and in Brazil.

Figure 3 depicts the progression of the fraction of P.1 genomes in the sample for the State of Amazon and for Brazil. In Brazil, the proportion of all E484K samples was nearly negligible between June 2020 and September 2020, emerging in October and hovering at slightly above 40% until January 2021. In the State of Amazonas, the E484K mutation was not detected until October 2020, with a steep and relatively linear rise from November 2020 onward, reaching 100% in January 2021. Between October 2020 and January 2021, the state of Amazonas experienced an overwhelming rise in the prevalence of E484K compared to Brazil overall. In both Brazil and the state of Amazonas, P.1 lineages were not detected until November 2020 and then increased progressively since December 2020. The rate of growth for P.1 lineage was more than two times faster in the State of Amazon compared to Brazil.

Social isolation and the subsequent prevalence of P.1 and E484K in Amazonas and Brazil

Table 2 shows the correlation between the SII of a given week and the prevalence of all P.1 strains in the following weeks. In the state of Amazonas, the SII of a given week was positively, significantly, and strongly (r > 0.7) correlated with the prevalence of the P.1 lineage in the four following weeks and was moderately correlated (0.6 < r < 0.7) in the fifth and sixth weeks after the SII on a given week. Distinctly from the state of Amazonas, overall Brazil demonstrated a weaker and shorter positive correlation between SII and P.1 lineage, only presenting one week after the SII on a given week.

**Table 2:** Spearman correlations between the mean of the Social Isolation Index in a given week and the fraction of all P.1 variants in the eight following weeks, in the state of Amazonas and in whole Brazil.

Weeks Later	State of Amazonas				Whole Brazil			
	Rho	R^2^	p-Value	N	Rho	R^2^	p-Value	N
0	0.77	59.5%	<0.01	17	0.35	12.3%	0.04	34
1	0.78	60.7%	<0.01	17	0.32	10.0%	0.07	33
2	0.77	59.1%	<0.01	16	0.34	11.2%	0.06	32
3	0.75	56.2%	<0.01	16	0.22	4.7%	0.24	31
4	0.76	57.5%	<0.01	16	0.16	2.7%	0.39	30
5	0.65	42.5%	0.01	16	-0.09	0.8%	0.64	29
6	0.65	42.9%	0.01	16	-0.11	1.3%	0.57	28
7	0.40	16.4%	0.12	16	-0.21	4.2%	0.30	27
8	0.09	0.8%	0.75	16	-0.38	14.4%	0.06	26

Table 3 shows the correlation between the SII on a given week and the prevalence of E484K variations in the following weeks. In the State of Amazonas, the SII in a given week was positively, moderate-to-intense, and significantly associated with the prevalence of E484K variants within the six weeks following the SII of a given week. Paradoxically, in overall Brazil, correlations between the E484K variants and the SII were significantly negative between four to eight weeks after the SII of a given week.

**Table 3:** Spearman correlations between the mean of the Social Isolation Index in a given week and the fraction of all E484K variants in the eight following weeks in the state of Amazonas and in whole Brazil.

Weeks Later	State of Amazonas				Whole Brazil			
	Rho	R^2^	p-Value	N	Rho	R^2^	p-Value	N
0	0.76	57.7%	<0.01	17	-0.03	0.1%	0.87	34
1	0.64	40.9%	0.01	17	-0.13	1.6%	0.49	33
2	0.69	48.3%	<0.01	16	-0.19	3.7%	0.29	32
3	0.70	49.5%	<0.01	16	-0.26	6.8%	0.16	31
4	0.61	36.7%	0.01	16	-0.42	17.5%	0.02	30
5	0.52	27.0%	0.04	16	-0.55	30.4%	<0.01	29
6	0.56	31.2%	0.02	16	-0.51	25.9%	0.01	28
7	0.31	9.5%	0.25	16	-0.58	33.4%	<0.01	27
8	0.08	0.6%	0.77	16	-0.58	33.9%	<0.01	26

The growth in P.1 and E484K in the state of Amazonas and Brazil according to the level of social isolation

Tables 4, 5 show the correlation between the prevalence of all P.1 lineage and E484K variants, respectively, and the number of COVID-19 deaths in the following weeks in the state of Amazonas and in whole Brazil. In the state of Amazonas, the correlations between the prevalence of both P.1 lineage and E484K variants, and the number of COVID-19 deaths in the following weeks were positive, statistically significant and strongest in the two weeks following the detection of the mutations (Spearman Rho=0.71; R^2^=51.1%; p<0.01; n=14). Conversely, for whole Brazil, there were no statistically significant associations between the prevalence of P.1 and further COVID-19 deaths, whereas there were statistically significant negative correlations in the four weeks following the detection of E484K variants (Spearman Rho=-0.61; R^2^=37.5%; p<0.01; n=32).

**Table 4:** Spearman correlations between the mean of the fraction of all P.1 variants and the total amount of weekly deaths in the eight following weeks in the state of Amazonas and in whole Brazil.

Weeks Later	State of Amazonas				Whole Brazil			
	Rho	R^2^	p-Value	N	Rho	R^2^	p-Value	N
0	0.51	26.0%	0.05	15	-0.04	0.2%	0.82	32
1	0.37	13.6%	0.18	15	-0.02	0.0%	0.91	31
2	0.71	51.1%	<0.01	14	0.01	0.0%	0.94	30
3	0.50	25.1%	0.08	13	0.01	0.0%	0.94	29
4	0.40	15.7%	0.20	12	0.01	0.0%	0.95	28
5	0.50	25.1%	0.12	11	0.00	0.0%	1.00	27

**Table 5:** Spearman correlations between the mean of the fraction of all E484K variants and the total amount of weekly deaths in the eight following weeks in the state of Amazonas and in whole Brazil.

Weeks Later	State of Amazonas				Whole Brazil			
	Rho	R^2^	p-Value	N	Rho	R^2^	p-Value	N
0	0.44	19.5%	0.10	15	-0.61	37.5%	<0.01	32
1	0.30	9.2%	0.27	15	-0.57	32.2%	<0.01	31
2	0.61	37.1%	0.02	14	-0.51	25.6%	<0.01	30
3	0.30	9.0%	0.32	13	-0.40	16.3%	0.03	29
4	0.26	6.8%	0.41	12	-0.29	8.6%	0.13	28
5	0.00	0.0%	1.00	11	-0.16	2.4%	0.44	27
6	0.41	16.6%	0.24	10	-0.01	0.0%	0.97	26
7	0.18	3.1%	0.63	10	0.11	1.1%	0.61	25
8	0.41	17.0%	0.24	10	0.20	4.0%	0.35	24

**Figure 4:**
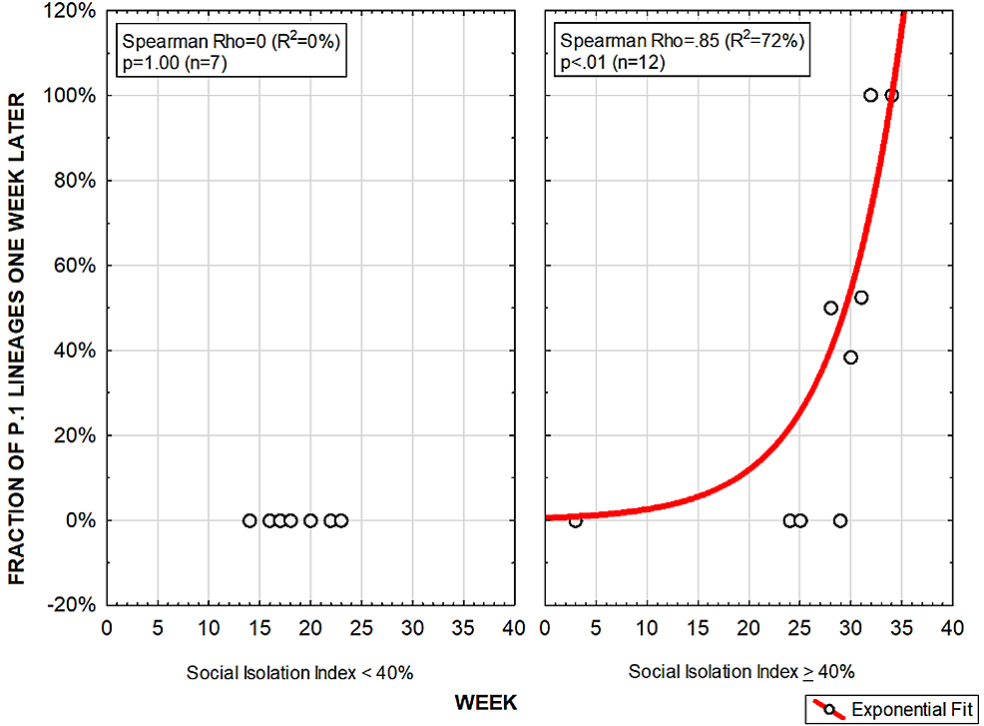
Social Isolation Index and prevalence of the P.1 lineage in the following week, when below 40% and when above 40%, in the state of Amazonas, Brazil.

**Figure 5:**
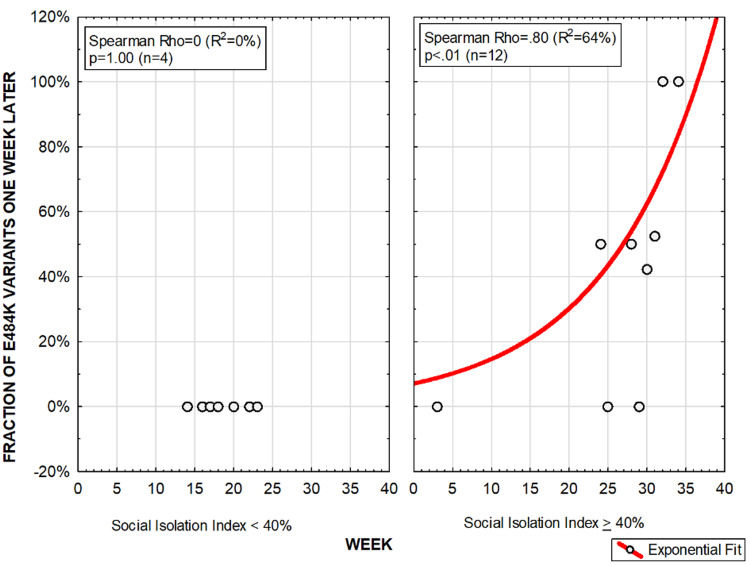
Social Isolation Index and prevalence of the E484K variant in the following week, when below 40% and when above 40%, in the state of Amazonas, Brazil.

Figures 4, 5 show the association between SII and prevalence of the P.1 lineage and E484K variants in the following week in the state of Amazonas (Brazil), respectively. For both correlations with P.1 lineage and E484K variants, figures were shown for SII below 40% and above 40%. When SII was found to be below 40%, P.1 lineage or E484K variants were not detected. When SII was above 40%, apparently exponential positive correlations between SII and prevalence of both P.1 lineage and E484K variants were observed.

**Figure 6:**
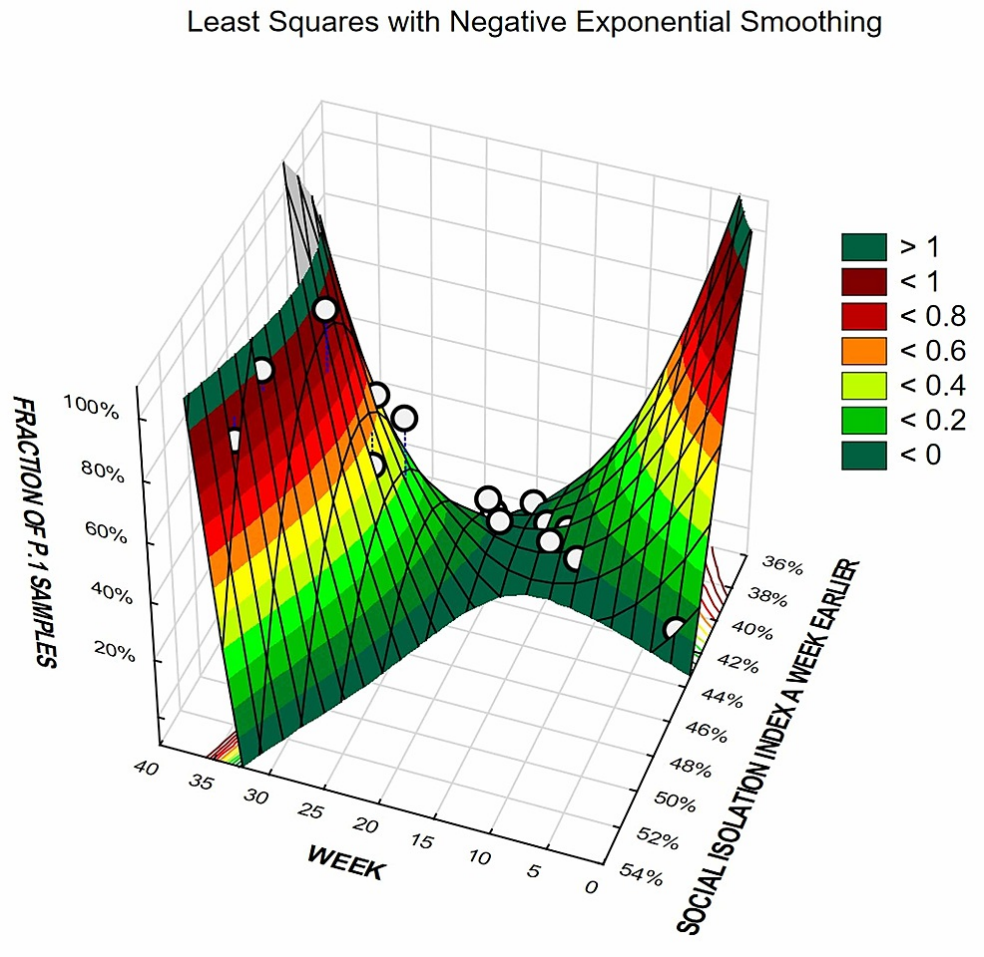
Social Isolation Index and prevalence of the P.1 lineage in the following week, according to the level of SII, for the state of Amazonas.

**Figure 7:**
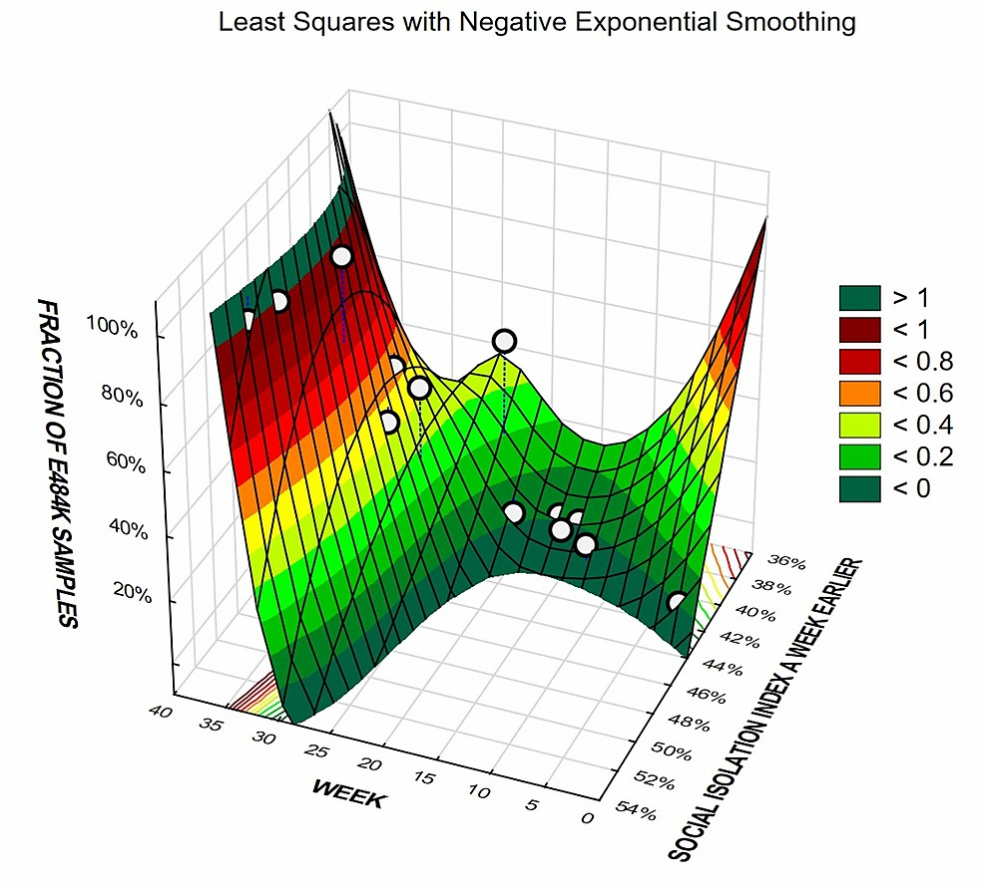
Social Isolation Index and prevalence of E484K variants in the following week, according to the level of SII, for the state of Amazonas.

Figures 6, 7 illustrate temporal progression of the prevalence of P.1 lineage and E484K variant, respectively, according to the level of SII in the previous week, through 3D surfaces. A sort of “twisted” patterns were detected for both P.1 lineage and E484K variants change substantially according to the level of SII in the previous week, in which the nadir seems to be when SII value is approximately 40%.

## Discussion

The second significant outbreak in the State of Amazonas, Brazil, was initiated in December 2020 and coincided with the emergence of a new sub lineage of the SARS-CoV-2 derived from B1.1.28, initially termed as sublineage P.1, and now recognized as being a full new lineage. The P.1 lineage was first detected by Japanese sanitary authorities in the Airport of Tokyo in four travelers returning from Manaus in January 6th, 2021 [7-11]. Genomic analyses of the SARS-CoV-2 revealed the presence of 12 new non-synonymous mutations, with 10 of them located in the spike (S) protein. Three of these mutations - K4017T, E484K, and N501Y - are present in the receptor-binding domain, which is critical for ligand-receptor interaction, and were similar to the three mutations detected in the South African B1.351 lineage [12]. Five of the 12 mutations detected in the P.1 lineage are located in the N-terminal domain, which, although not directly involved in the recognition of cell receptor, may induce substantial effects in the ligand-receptor surface through allosteric interactions. Given that a specific mutation could have different effects on receptor binding affinity and the capacity for immune evasion, we speculate that not all substitutions are equally relevant for SARS-CoV-2 infectivity. Specifically, E484K mutations seem particularly critical because its shifts the main site for interaction with the human membrane-attached angiotensin conversing enzyme-2 (ACE-2) [10-12].

We have conducted an analysis attempting to correlate 773 genomic samples from Brazil, of which 77 were from the State of Amazonas, with SII. Amazonas is among the states with the highest levels of SII in Brazil, consistently above the country's average, and has experienced a rapid increase in SII from October 2020 to January 2021.

Amazonas has also one of the largest sizes of households [13]. The combination of high SII and large size of households could have been determinant for the development of new SARS-CoV-2 variants. In the present study, SII was found to be positively associated with a substantial rise in the prevalence of these new variants in the following weeks. However, this correlation could only be observed when SII was above 40% (from November 2020 to January 2021), suggesting that the SARS-CoV-2 ability to mutate was dependent on high levels of SII in the state of Amazonas, Brazil. Data regarding level of SII and viral ability to mutate was consistent since both P.1 lineage and E484K variants were in general correlated with SII in a similar manner. The consistent correlations between SII and speed of emergence of new SARS-CoV-2 variants reinforce the hypothesis that forced prolonged cohabiting may boost viral ability to generate mutation that may eventually lead to immune evasion and increased infectivity.

While the occurrence of P.1 and E484K variants were both positively correlated with SII in the state of Amazonas, neutral and negatively correlations were detected in Brazil. The distinct behaviors identified in the present analysis could be related to the level of heterogeneity of the lineages and number of acquisition events. While in Brazil, variants were mostly heterogeneous, reflecting a large number of independent acquisition events, E484K harboring mutations in detected in the state of Amazonas were mostly derived from a highly homogeneous P.1 lineage [14], possibly as a consequence of fewer multiple-mutation events possibly related to higher levels of SII.

Although the exact reasons that justify the distinct behaviors are yet to be fully clarified, the key meaning is that stay-at-home orders may not necessarily lead to reduction in the number of further cases, particularly in the long run, as high SII may induce emergence of new SARS-CoV-2 variants, among which some may present increased transmissibility and pathogenesis.

Our findings could be explained, at least in part, by overdispersion coefficient (k) characteristics of SARS-CoV-2, which has been consistently set at 0.1 [15,16], i.e., approximately 10% of infected subjects are responsible for more than 80% of all SARS-CoV-2 infections. Effective protective strategies should focus on avoidance of these not prevailing but highly relevant “superspreading events”.

In cell models, culture-derived adaptive viral mutation can substantially increase overall viral replication capacity, of up to two logs higher viral concentration after serial passaging [17]. The emergence of SARS-CoV-2 mutations in Brazil seems to have occurred concurrently with the increase in the levels of SII. From the data presented, there is sufficient plausibility to hypothesize that the new viral lineage emerged from a forced viral evolution due to increased SII in an environment of a large number of households, which is the peculiar case of the state of Amazonas, Brazil.

Since the present findings should be considered as preliminary data and require further confirmation, these findings should not be used to drive further policies against social isolation and distancing. However, we suggest that the implementation of social isolation and distancing policies should start to consider the particular effects of SII in specific regions in a more comprehensive manner not only in terms of further cases and COVID-19 deaths but also considering the consequences of potential increased speed of emergence of new SARS-CoV-2 variants.

Limitations of the study

The SARS-CoV-2 genomes analyzed in the present study were not randomly chosen or resulted from controlled stratifications, does not reflect the number of cases of each period analyzed, and is not representative of the population analyzed.

The present communication does not have the power to establish causality. Indeed, other factors than SII may influence the emergence of new SARS-CoV-2 variants, in particular for the E484K mutations [17]. However, at the best of our knowledge, this is the first attempt to correlate SII and emergence of E484K variants, including the new lineage P.1.

## Conclusions

The results of this study indicate that SII above 40% is associated with the emergence of SARS-CoV-2 E484K variants and P.1 lineage in the state of Amazonas, Brazil, whereas this correlation has not been found in overall Brazil during the period analyzed.
